# Automated Image Analysis of the Host-Pathogen Interaction between Phagocytes and *Aspergillus fumigatus*

**DOI:** 10.1371/journal.pone.0019591

**Published:** 2011-05-05

**Authors:** Franziska Mech, Andreas Thywißen, Reinhard Guthke, Axel A. Brakhage, Marc Thilo Figge

**Affiliations:** 1 Research Group Applied Systems Biology, Leibniz Institute for Natural Product Research and Infection Biology – Hans-Knöll-Institute (HKI), Jena, Germany; 2 Department of Molecular and Applied Microbiology, Leibniz Institute for Natural Product Research and Infection Biology – Hans-Knöll-Institute (HKI), Jena, Germany; 3 Friedrich Schiller University Jena, Jena, Germany; 4 Research Group Systems Biology/Bioinformatics, Leibniz Institute for Natural Product Research and Infection Biology – Hans-Knöll-Institute (HKI), Jena, Germany; New York State Department of Health and School of Public Health, University at Albany, United States of America

## Abstract

*Aspergillus fumigatus* is a ubiquitous airborne fungus and opportunistic human pathogen. In immunocompromised hosts, the fungus can cause life-threatening diseases like invasive pulmonary aspergillosis. Since the incidence of fungal systemic infections drastically increased over the last years, it is a major goal to investigate the pathobiology of *A. fumigatus* and in particular the interactions of *A. fumigatus* conidia with immune cells. Many of these studies include the activity of immune effector cells, in particular of macrophages, when they are confronted with conidia of *A. fumigus* wild-type and mutant strains. Here, we report the development of an automated analysis of confocal laser scanning microscopy images from macrophages coincubated with different *A. fumigatus* strains. At present, microscopy images are often analysed manually, including cell counting and determination of interrelations between cells, which is very time consuming and error-prone. Automation of this process overcomes these disadvantages and standardises the analysis, which is a prerequisite for further systems biological studies including mathematical modeling of the infection process. For this purpose, the cells in our experimental setup were differentially stained and monitored by confocal laser scanning microscopy. To perform the image analysis in an automatic fashion, we developed a ruleset that is generally applicable to phagocytosis assays and in the present case was processed by the software Definiens Developer XD. As a result of a complete image analysis we obtained features such as size, shape, number of cells and cell-cell contacts. The analysis reported here, reveals that different mutants of *A. fumigatus* have a major influence on the ability of macrophages to adhere and to phagocytose the respective conidia. In particular, we observe that the phagocytosis ratio and the aggregation behaviour of *pksP* mutant compared to wild-type conidia are both significantly increased.

## Introduction

During the last decade automated image analysis became a very popular and widely-used tool in biology combined with sophisticated microscopy techniques, e.g. confocal laser scanning microscopy [1]. One application among others is the acquisition and subsequent analysis of images to determine phagocytosis ratios. A phagocytosis assay is an immunological method used to study the ability of phagocytes to detect and engulf foreign particles or organisms. A great advantage of this technique is the possibility of using different fluorescent dyes and thereby to distinguish between different cellular structures or cell types. Thus, images of cells labelled with different dyes are acquired for further investigation. Such a procedure is also often employed to study infection processes with the fungus *Aspergillus fumigatus*. This ubiquitous saprophytic mold is the most prevalent airborne opportunistic pathogenic fungus. *A. fumigatus* produces conidia during asexual reproduction as the common reproductive form. Every day healthy humans inhale hundreds of conidia without getting infected [2]–[4]. By contrast, in immunocompromised patients *A. fumigatus* can cause invasive pulmonary aspergillosis (IPA) that results in mortality rates of about 30–95% [5]–[7]. Therefore, fungal infections and their impact on the human immune system are a major issue in current research [2], [8]–[10] and the analysis of various mutants is of great importance to get deeper insight into the pathogenicity mechanisms of this fungus [5]. Since the proper recognition, adherence and ingestion of inhaled conidia by phagocytes represent critical steps in the infection process of *A. fumigatus*, the determination of phagocytosis ratios provides crucial insights into infection mechanisms. Until now, a variety of different protocols exists to study phagocytosis on the basis of differential fluorescent staining procedures and subsequent image data analysis [11], [12]. In the present, phagocytosis assays cells were differentially stained with fluorescent dyes and visualised with a confocal laser scanning microscope, leading to the discrimination between macrophages, internalised conidia and non-internalised conidia.

Evaluation of the acquired image data is typically carried out manually, which is very time-consuming, error-prone and the bottleneck for analysis [13], [14]. Therefore, digitalisation of the images is a prerequisite for automated image analysis which in turn increases the amount of data that can be analysed. Here, we combined confocal laser scanning microscopy with automated image analysis by a procedure that is knowledge-based, quantitative and adaptive to phagocytosis assays of any other *A. fumigatus* mutant.

**Figure 1:**
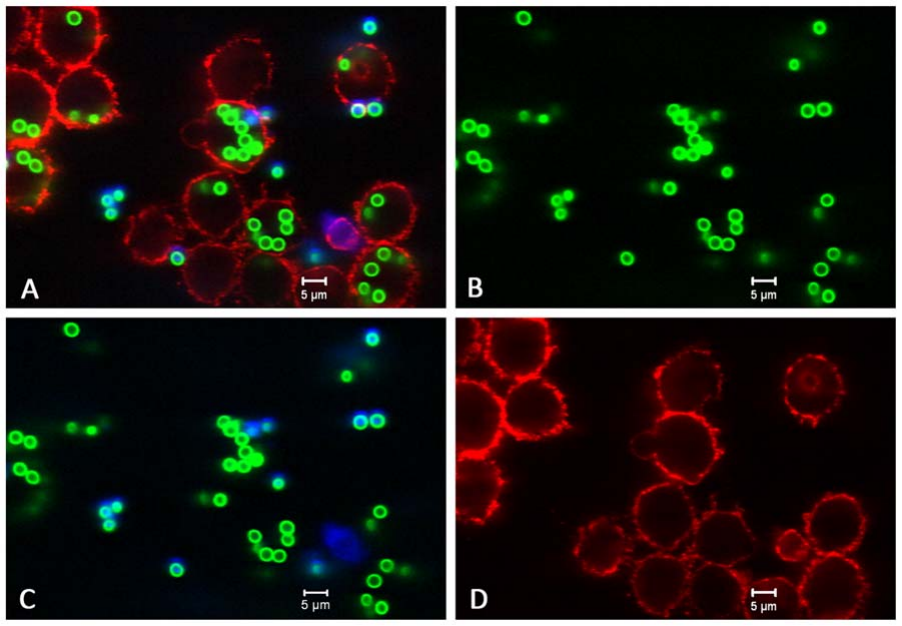
Original image of the phagocytosis assay. (A) Overlay of all layers showing all conidia and macrophages. (B) Green layer (FITC) with all conidia. (C) Blue layer (Calcoflour white) with exterior conidia. (D) Red layer (Cy5) with macrophages.

Currently, there are various image analysis methods and imaging tools available (for an overview see [15], [16]). In every application, image analysis comprises three parts: pre-processing, segmentation and classification. Pre-processing includes noise reduction and quality enhancing filtering, e.g. by Gaussian blur [17]. Comparison of the segmentation processes reveals the largest differences of these methods. Segmentation divides the image into multiple meaningful segments and the background. It is usually pixel-based, i.e. based on thresholding, gradients and pixel operations. A popular pixel-based segmentation method, which has proved to be useful on separating clustered objects, is the so-called watershed algorithm [18], [19]. It is either applied to the original image or to the image gradient [20]. Due to the intensity variations within clustered objects, watershed segmentation will almost always lead to over-segmentation. Existing merging methods to reduce over-segmentation often fail, due to a great amount of manual work to find appropriate border seeds for each image object from which a curve can be calculated along a strong object border [21]. Thus, these approaches only give sufficient results for well defined and homogenous objects with strong edges [13]. However, as can be seen in Fig. 1, this does not apply to fluorescence-stained conidia together with macrophages for the following reasons: (i) conidia and macrophages are often clustered and attached to each other, (ii) both cell types show variations in their internal and in-between intensities, and (iii) the background is variable [22]. Therefore, we explored an entirely different approach, which is context-based to meet all of the aforementioned challenges. The Cognition Network Technology (CNT) used in the present work represents an object-oriented image analysis approach that models human cognitive processes. Implemented in the Definiens Developer XD platform [23], it was originally developed as a tool for analysing satellite images. Recently, it was also applied to histopathology, magnetic resonance imaging and high-throughput chemical genetics [24]–[26]. With CNT, an understanding of the present images was built iteratively, including the identification, i.e. segmentation and classification of objects through their characteristics, shapes, relationships to other objects and the context in which they occur. The object features and properties were captured in a self-organising, semantic, self-similar hierarchical network to simplify access to them [27]. In this way, segmentation and classification were carried out repetitively rather than sequentially which led to a hierarchy of the cell types, e.g. conidia and macrophages on one level and below different types of conidia (non-adherent exterior, adherent, phagocytosed) with all their interrelations. The instructions to form such a network are written in a meta-language (Cognition Network Language) and the combination of all commands forms a ruleset that does not only solve the problem at hand but is generally applicable to phagocytosis assays and allows for the efficient analysis of high-throughput microscopy measurements.

## Materials and Methods

### Experimental Procedures

#### *A. fumigatus* strains and growth condition

*A. fumigatus* wild-type ATCC 46645 and the *pksP* mutant strain were cultivated on *Aspergillus* minimal medium (AMM) agar plates with 1% (wt/vol) glucose at 37°C for 5 days. AMM consisted of 70 mM NaNO_3_, 11.2 mM KH_2_PO_4_, 7 mM KCl, 2.1 mM MgSO_4_ and 1 µl/ml trace element solution (pH 6.5). The trace element solution consisted of 1 g FeSO_4_·7H_2_O, 8.8 g ZnSO_4_·7H_2_O, 0.4 g CuSO_4_·5H_2_O, 0.15 g MnSO_4·_ 4H_2_O, 0.1 g NaB_4_O_7_·10H_2_O, 0.05 g (NH_4_)_6_Mo_7_O_24_·4 H_2_O, and double-distilled water (ddH_2_O) to 1,000 ml [28]. Conidia were harvested in sterile 0.9% (wt/vol) NaCl, 0.1% (vol/vol) Tween20.

FITC-labelling of conidia was performed with 0.1 mg/ml FITC (Sigma) in 0.1 M Na_2_CO_3_ at 37°C for 30 min. Labelled conidia were washed three times with PBS, 0.1% (vol/vol) Tween20. Conidia concentration was determined using a Thoma chamber.

#### Phagocytosis experiments

Murine alveolar MH-S macrophages (ATCC CRL-2019) were cultivated in RPMI1640 medium supplemented with 10% (vol/vol) FCS, 2 mM ultraglutamine 1 and gentamycin. For infection experiments, MH-S macrophages were seeded on glass cover slips in 24 well plates at a density of 5×10^5^ cells per well and allowed to grow adherently over night. Following washing with prewarmed medium, FITC-labelled conidia were added at a multiplicity of infection of 5. The infection experiment was synchronised for 30 min at 4°C. Unbound conidia were removed by washing with pre-warmed medium and phagocytosis was initiated by shifting the coincubation to 37°C in a humidified CO_2_ incubator. After 1 h the phagocytosis was stopped by washing with ice-cold PBS. Labelling of extracellular conidia was performed by incubation with PBS, 0.25 mg/ml calcofluor white (Sigma) for 30 min at 4°C to avoid further ingestion. The cells were washed twice with PBS and fixed with 3.7% (vol/vol) formaldehyde/PBS for 15 min followed by two washes with PBS. To avoid non-specific binding of antibodies, cells were incubated in PBS, 3% (vol/vol) BSA for 30 min. Macrophages were labelled with a monoclonal rat anti-CD9 antibody (1∶200) as primary antibody and a goat anti-rat-IgG-Cy5 antibody (1∶200). Microscopic photographs were taken on a Zeiss LSM 5 Live confocal laser scanning microscope.

For statistical reproducibility two biological replicates and in each case two technical replicates were made and analysed for each strain.

### Automated Image Analysis

In this section, we describe the ruleset of the image pre-processing and the segmentation and classification process for the automated image analysis. The software Definiens Developer XD and Definiens eCognition® Server XD was implemented on one core of a SUN Fire X4600 Server M2 (8 CPUs with 4 cores each, 2.3 GHz AMD Opteron, 64 GB memory).

**Figure 2:**
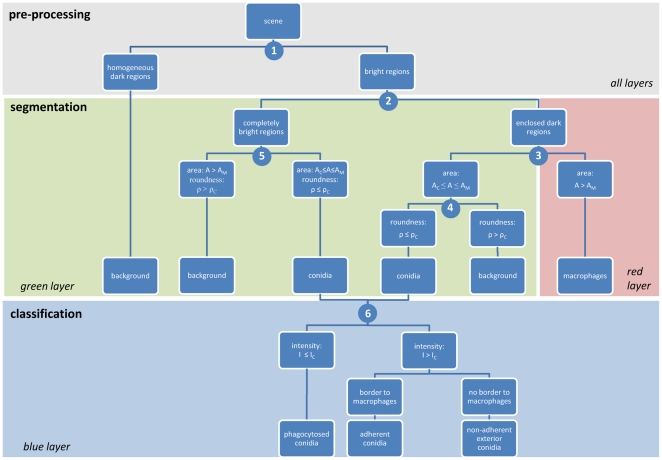
Schematic representation of the rulset-based segmentation and classification process for the automated image analysis on the three colour layers containing all conidia (green layer), exterior conidia (blue layer), and macrophages (red layer). Encircled numbers denote split points.

Each image is built of three distinct layers, one for each fluorescent label. As is shown in Fig. 1, layer one shows all conidia stained in green (FITC), layer two all non-phagocytosed conidia with a blue dye (calcofluor white), and layer three macrophages in red (Cy5). The ruleset of the automated image analysis is summarised in Fig. 2. The implementation of this ruleset in the software Definiens Developer XD is provided in File S1.

#### Pre-processing

**Figure 3:**
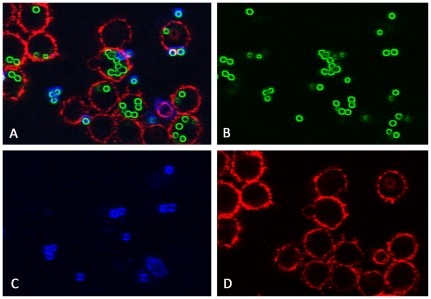
Image of the phagocytosis assay as in **Fig. 1** after pre-processing. (A) Overlay of all layers showing all conidia and macrophages. (B) Green layer (FITC) with all conidia. (C) Blue layer (Calcoflour white) with exterior conidia. (D) Red layer (Cy5) with macrophages.

The size of the images is 1024×1024 pixel with a distance of 0.2 µm between two pixels. All three distinct layers were smoothed with a 3×3 Gauss filter of Gaussian width σ = 1 to reduce noise. Afterwards an edge-detection filter was applied to enhance object boundaries. This filter assigns to every pixel the maximal intensity value of its 5×5 pixel neighbourhood. The resulting image is shown in Fig. 3 and has pixels with high intensity values where there are distinct changes of the intensities in the original image (Fig. 1). No further pre-processing was necessary as the successive application of both filters led to images with enhanced features, i.e. high object boundary values and low background values, which simplify identification and segmentation in the subsequent segmentation and classification process.

#### Segmentation and Classification

The conidia as well as the macrophages were stained by labelling different structures on their respective surfaces. Therefore, all cells appear with a bright border and are darker at the inside. Thus, the envelopes of the cells represent the regions of interest (ROI). The first step of the process (split point 1 in Fig. 2) divides the images into these ROI and the background by applying an automatic threshold method on each filtered layer. This method comprises a combination of a histogram and homogeneity measurement, such that intensity differences and heterogeneity increase to a maximum between the resulting pixel-subsets. Dark and homogeneous subsets form the background. The remaining subsets are the ROI and either consist of bright pixel regions or of regions heterogeneously structured containing dark and bright pixels. In the second step (split point 2 in Fig. 2), ROI are split according to their intensity values of the green layer into completely bright regions and regions that enclose dark areas.

At the third split point the ruleset distinguishes regions that enclose dark areas by their size using that macrophages and conidia have clearly distinct areas, which were computed from our experiments to correspond to 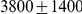 pixels and 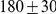 pixels, respectively. Accordingly, we set the threshold area to 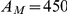 pixels which translates into the threshold diameter 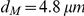 for a circular cell. Areas exceeding 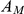 are classified as macrophages, if their intensity values on the red layer are higher than the limit that was calculated by the aforementioned automatic threshold method. Objects that have areas smaller than 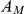 are labelled as conidia, except if their areas are smaller than 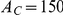 pixels. This area corresponds to the diameter 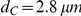 for a circular cell and objects below this size are considered to be artefacts of the initial segmentation. They are merged with the background (not shown in Fig. 2). The conidia undergo a further morphological operation by pixel-based growing into the bright borders of the ROI. This growing is based on layer intensities and shape, i.e. roundness, in the following way. The roundness 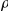 of an object is measured by the similarity of its shape to a circle. Approximating the object by an outer and an inner ellipse, this value is specified as the difference between the length of the major axis of the outer ellipse 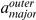 and that of the minor axis of the inner ellipse 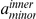:
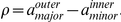



Thus, for 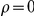 pixel the object corresponds to a circle. In the developed ruleset, the pixel set of a conidium is allowed to grow as long as the added pixel intensities are brighter than the previous object border pixels and under the condition that the roundness remains under a threshold value 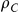. It turns out that for 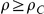 with 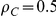, objects have such an irregular shape that they should be considered as artefacts that are merged with the background (split point 4 in Fig. 2). Completely bright regions are classified as conidia only if their size is between 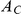 and 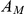 and their roundness is below 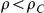 (split point 5 in Fig. 2). Otherwise, bright regions are classified as artefacts. Thus, the resulting objects of these first rule-based steps clearly define the separation of conidia and macrophages.

Next, for cell counting the ruleset distinguishes between phagocytosed, adherent and non-adherent exterior conidia (split point 6 in Fig. 2). Using the context-based approach all relations between neighbouring objects are stored in the network-hierarchy established by the ruleset. The distinction between different conidia memberships has to be specified based on the blue layer, because due to the staining (see section on Experimental Profocedures) only adherent and non-adherent exterior conidia appear in blue. Conidia objects are classified depending on their average intensity 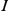 in the range of integer values from 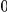 to 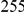. We find that for intensities above a critical value, 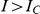 with 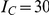 for the blue layer, conidia can be successfully classified as exterior conidia, whereas objects with lower intensity values, 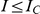, are artefacts that may arise by reason of halo effects from adjacent conidia. Objects that are classified as exterior conidia and share a border with macrophages are re-labelled as adherent conidia.

After this ruleset-based image data processing, the features obtained for all four labelled classes (macrophages, phagocytosed conidia, exterior conidia that can be either adherent or non-adherent) were exported and used for subsequent analyses, e.g. area in pixel, layer intensity and number of neighbours of each object as well as class membership of every object. Finally, the number of cells per class is calculated to perform statistical analyses and validation procedures.

## Results

**Figure 4:**
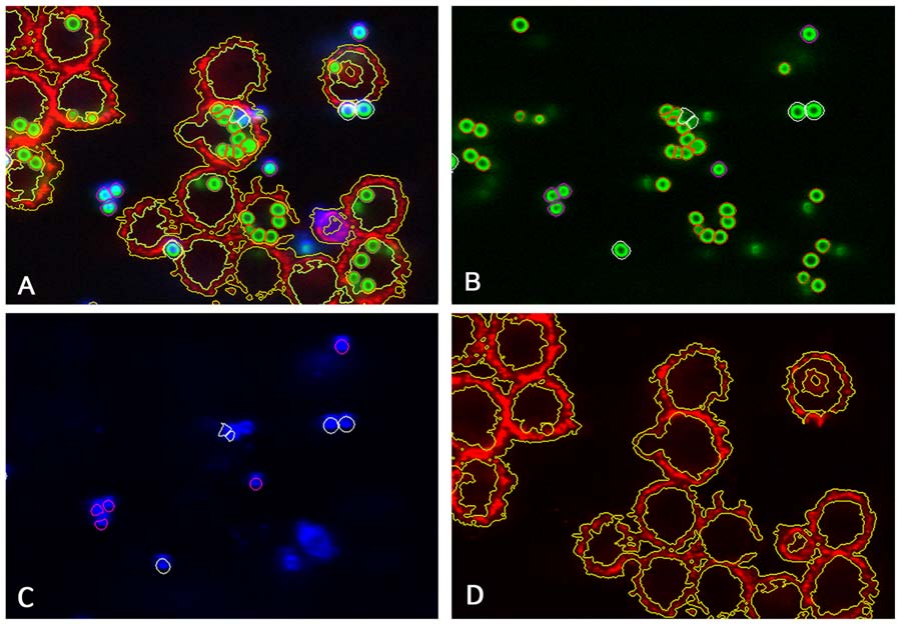
Image of the phagocytosis assay as in **Fig. 2** after segmentation and classification. (A) Overlay of all layers showing all conidia and macrophages. (B) Green layer (FITC) with exterior non-adherent conidia encircled with magenta colour, adherent conidia encircled with white colour, and interior conidia encircled with orange colour. (C) Blue layer (Calcoflour white) with exterior and adherent conidia encircled with the same colours as in (B). (D) Red layer (Cy5) with macrophages encircled with yellow colour.

As a proof of principle, we demonstrate that the automated context-based image analysis successfully verifies image data independent of the experimenter. The developed ruleset was tested on confocal laser scanning microscopy images derived from phagocytosis assays of conidia from *Aspergillus fumigatus* wild type (ATCC 46645) and the *pksP* mutant lacking DHN-melanin. The analysis was based on 40 images of the wild type and 40 images of the mutant. The typical computation time per image is between 20 and 30 seconds, depending on the number of objects to be identified. Input parameters were set carefully for a set of five test images and then applied to the residual 75 images for validation. Only four input parameters were required: threshold values for conidium and macrophage areas (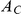 and 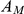), maximum conidium roundness (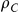) and threshold intensity for conidia (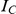). The images in Fig. 3 are the result of pre-processing the original images in Fig. 1 and the results of the subsequent segmentation procedure on these images are shown in Fig. 4. The number of objects identified as conidia per image was between 17 and 225, indicating a broad variance of the conidial distribution. A description of the complete workflow comprising the analysis is provided in File S2.

### Validation of the Automated Image Analysis

The results obtained from the automated analysis were validated by separately comparing segmentation and classification with a careful analysis by human experts as a reference. Table 1 displays the validation outcome for the segmentation process. For each image, the number of incorrect segmented conidia (false positives segmentation, 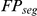) and conidia which were not detected at all (false negatives segmentation, 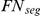) were counted. False positives arose from over-segmentation. An over-segmented object was wrongly split into two or more smaller objects due to high intensity values in inner-object regions when applying the edge-detection filter (see section on Automated Image Analysis). In contrast, false negatives are due to conidia intensity values that are too low on both image layers (blue and green) to allow for differentiation from background noise. Subtracting 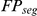 from the number of segmented objects yielded the number of conidia per image (true positives segmentation, 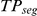) which were correctly segmented. Furthermore, the 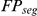 rate and 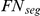 rate are computed as the ratio of 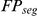 or 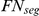 to the total number of segmented conidia (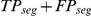) relative to the manual revision.

**Table 1:** Results for segmentation of conidia.

	no. of segmented	*FP_seg_*	*FN_seg_*	*TP_seg_*	
type	conidia	*FP_seg_* rate	*FN_seg_* rate	*S_seg_*	*P_seg_*
wild type	2712	62	73	2650	
		2.28%	2.68%	97.32%	97.71%
*pksP*	3803	157	218	3646	
		4.06%	5.64%	94.36%	95.87%
Σ	6515	218	290	6325	
		3.30%	4.38%	95.62%	96.67%

*FP_seg_* and *FN_seg_* correspond to over- and undersegmentation. Sensitivity is denoted by *S_seg_* and precision by *P_seg_*.

*TP_seg_* denotes correctly segmented conidia.

The performance of the segmentation, of conidia was measured using two metrics. First, the precision (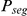) being the ratio of the number of correctly segmented conidia (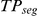) to the total number of segmented conidia (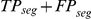):
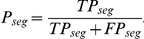



Second, the sensitivity (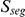) being the ratio of the number of truly segmented conidia (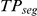) to the total number of observable conidia in the images (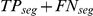):
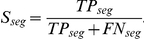



Precision and sensitivity of the segmentation reached up to 96% and 97%, respectively (Table 1).

The segmentation process was followed by a classification of the segmented conidia into corresponding classes namely phagocytosed conidia, adherent conidia and non-adherent exterior conidia. This discrimination is based on the roundness parameter 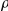 and the average intensity 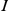 (see section on Automated Image Analysis). Table 2 shows the number of conidia per class. The false positive rate (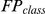 rate) denotes the number of misclassified conidia to the number of correct classifications. Misclassifications occurred, e.g. if conidia in the green layer happen to have intensity values below 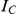 in the blue layer (non-adherent exterior conidia only). Thus, a conidium was labelled as an inner conidium even though it is an adherent conidium. These conidia are slightly bright specks that may be detectable by human experts. On the other hand, accurate classification is always subjected to the particular expertise. 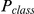 and 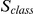 were calculated as well for the classification part and yielded 99% for both values. Furthermore, combining segmentation and classification, we present the values for 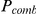 and 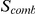, that are calculated from 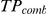, 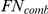 and 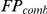, as given in Table 3.

**Table 2:** Results for specific classification of conidia into corresponding classes.

		classification into			*FP_class_*	*FN_class_*	*TP_class_*	
type	no. of classified conidia	phagocy-tosed conidia	adherent conidia	non-adherent exterior conidia	*FP_class_* rate	*FN_class_ rate*	*S_class_*	*P_class_*
wild type	2712	1026	1465	221	63	60		2649
					2.32%	2.22	97.79%	97.68%
*pksP*	3803	2348	1168	287	17	17		3786
					0.47%	0.47	99.55%	99.55%
Σ	6515	3374	2633	508	80	77		6435
					1.23%	1.18%	98.82%	98.77%

*TP_class_* denotes correctly classified conidia. *FP_class_* indicates the number of misclassifications. *FN_class_* as the number of conidia not classified at all. Sensitivity is denoted by *S_seg_* and precision is denoted by *P_class_*.

**Table 3:** Combined results for segmentation and classificaiton of conidia.

	no. of segmented and classified	*FP_comb_*	*FN_comb_*	*TP_comb_*	
type	conidia	*FP_comb_* rate	*FN_comb_* rate	*S_comb_*	*P_comb_*
wild type	2712	125	133	2587	
		4.39%	4.68%	95.11%	95.39%
*pksP*	3803	174	235	3629	
		4.50%	6.08%	93.92%	95.43%
Σ		299	368	6216	
		4.54%	5.59%	94.41%	95.41%

*FP_comb_* denotes the combined number of segmentation errors and misclassifications. *FN_comb_* correspond undersegmentation and no classification. Sensitivity is denoted by *S_comb_* and precision by *P_comb_*.

*TP_comb_* refers to the number of correctly segmented and classified conidia.

Conidia are classified in two successive steps. We denote the first step by 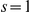, which comprises the differentiation into phagocytosed conidia and adherent exterior conidia according to their layer intensities. In the second step with 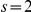, the distinction between adherent and non-adherent exterior conidia follows based on their spatial proximity to macrophages. Validation of the performance of the two-step classification was carried out separately by calculating two measures. The 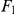-measure [29] is the harmonic mean of precision (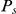) and sensitivity (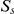),
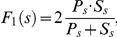
which takes values between 0 and 1. The higher the value of *F_1_* the better the classification. In the context of the two-step classification, 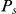 and 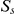 were calculated based on the classes which the classifier assigns to the conidia. The Matthews correlation coefficient (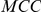) [30], [31] is a measure of the quality of two-class classifications. It can be used even if the classes are of very different sizes and takes into account true and false positives and negatives:
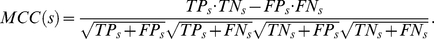



The value of 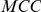 is between 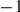 and 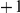. For 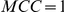, the classification is considered to be perfect, whereas 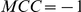 indicates an inverse classification. An intermediate value of 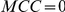 corresponds to a random classification. In this regard, the true and false positives and negatives were calculated based on the classes which were assigned to the conidia and are given in Table 4. For both measures, 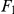 and 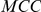, the ruleset-based classifier yields high values close to 1, which impressively demonstrate the high performance of the established ruleset (Table 4). In passing we note that there were no macrophages misclassified as conidia and vice versa.

**Table 4:** Validation results: Measures of classification performance.

	*classification step s* = 1 (inner vs. exterior conidia)	*classification step s* = 2 (adherent vs. non-adherent exterior conidia)
*TP_s_*	3302	2627
*FP_s_*	71	6
*FN_s_*	7	59
*TN_s_*	3203	518
*F_1_*	0.99	0.99
*MCC*	0.98	0.93

### Comparative Analysis for Two *A. fumigatus* Strains

**Figure 5:**
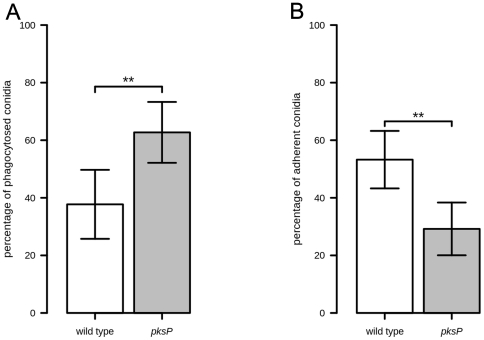
Results for phagocytosis and adhesion of wild-type and *pksP* conidia. (A) Comparison of the ratios of phagocytosis. (B) Comparison of the ratios of adhesion. **: *p*-value<0.01.

The two *A. funigatus* strains, ATCC and *pksP*, were chosen because Luther *et al.* previously demonstrated that the *pksP* mutant shows an increased phagocytosis ratio compared to the wild-type strain [32]. The ratio consists of the number of phagocytosed conidia to all macrophage-associated conidia. In our study, the mean of this ratio was found to be 66% higher for the *pksP* mutant conidia than for the wild-type conidia, as can be seen in Fig. 5 A. In Table 5 we provide a comparison of the manually and automatically obtained phagocytosis ratios to underline that the automated analysis revealed similar results compared to the manual analysis.

**Table 5:** Comparison of mean and standard deviation for the manual versus automatic results of phagocytosis ratios.

	manual		automated	
	mean	std	mean	std
wild type	34.96%	10%	37.73%	9.98%
*pksP*	66.52%	9%	62.72%	9.16%

Furthermore, we computed the adhesion ratio as the ratio of adherent conidia over all macrophage-associated conidia. In Fig. 5 B, it can be seen that the mean value of the adhesion ratio is 45% lower for the *pksP* mutant conidia than for the wild-type conidia. We determined whether these results are significant and started by testing whether these data fulfill the assumption of a normal distribution. To unravel possible deviations from the normal distribution, we applied Shapiro-Wilk, Anderson-Darling and Cramér-von Mises tests. All three tests verify the accuracy of the assumption for a normal distribution of the phagocytosis and adhesion ratios, as required for the application of the Welch *t*-test. The statistical analysis is summarised in Table 6 and reveals that the difference in both phagocytosis and adhesion ratios is highly significant (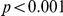).

**Table 6:** Statistical results for phagocytosis and adhesion ratios.

		*p*-value	
		phagocytosis ratio	adhesion ratio
**test of normality**			
Shapiro-Wilk	wild type	0.74	0.95
	*pksP*	0.87	0.41
Anderson-Darling	wild type	0.66	0.99
	*pksP*	0.84	0.55
Cramer-von Mises	wild type	0.66	0.99
	*pksP*	0.74	0.59
**test of significance**			
Welch t-test, *α*<0.001	wild type vs. *pksP*	7.189 10^−16^	2.2 10^−16^

**Figure 6:**
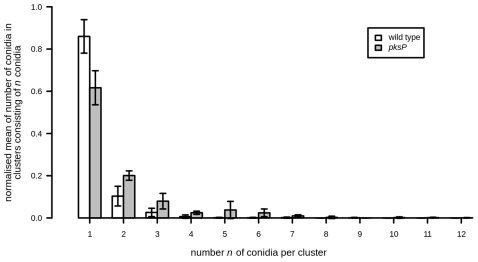
Comparison of the aggregation behaviour for the two strains. Normalised mean of conidia in clusters consisting of *n* conidia plotted as a function of the number *n*. *p-*value<0.01.

In addition to the determination of the phagocytosis and adhesion ratios our method provides results on the aggregation behaviour of conidia, as can be seen in Fig. 6. We found that on average 86% of the wild-type conidia appeared as isolated cells, whereas this fraction was only 62% for the *pksP* mutant conidia. It should be noted, however, that these cluster data are not normally distributed. Therefore, we applied the Wilcoxon signed-rank test and found that the difference concerning the aggregation of conidia between the two *A. fumigatus* strains is highly significant (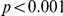). The increased aggregation of *pksP* mutant conidia is clearly visible from Fig. 6, where we show the normalised number of conidia in clusters consisting of *n* conidia as a function of *n*.

## Discussion

The major bottleneck in the analysis of phagocytosis of *A. fumigates* by immune effector cells is the labelling and counting of conidia, since to date this is still carried out manually. Fluorescence activated cell sorting (FACS) for determination of phagocytosis ratios might be time saving but leads to inaccurate results, because a clear discrimination of microbes being adherent to or ingested by the immune cells is not possible. Correspondingly, ratios of internalised microbes over all microbes associated with immune cells cannot be calculated from FACS. Microscopic analysis is still the standard to determine phagocytosis ratios in an accurate way. Various efforts were made to improve microscopic image analysis steps. However, up to now only the experimental procedures were modified without improving the most time consuming part of the manual processing, i.e. the analysis of the microscopic images. The method described here, represents a fully automated, context-based approach to perform this task. The developed ruleset allows for the segmentation and classification of the regions of interest (ROI), being image objects composed of macrophages and conidia, in an iterative way. Only four input-parameters in terms of threshold values are required, i.e. the areas 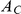 and 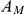, the roundness 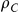 and the intensity 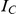. The method is able to identify conidia properly, even if they are clustered or have an asymmetrical shape. Only in particular cases with conidia being irregularly stained and showing high intensity variations, the ruleset may misidentify conidia, making either manual correction or a more specialised segmentation necessary by including further knowledge about the specific circumstances. In general, the application of our method enables the user to investigate a large amount of image data without the further need of intervention. This leads to the possibility to process more images and thereby to increase not only the amount of strains that can be tested but also the number of processed images per strain. In addition, we found that the time required for experts to classify the cells was about five minutes per image and, thus, ten times longer as compared to the automated image analysis. Nevertheless, the results of our method are comparable to the manual analysis by experts, with the advantage of avoiding biases due to human observers.

Our validation of the segmentation and classification results reflects that the number of false positives by reason of misclassification is quite acceptable. High 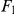-scores indicate a high classifier accuracy and demonstrate the ruleset as being able to clearly differentiate between the different types of conidia. Additional consideration of true negatives by the use of the Matthews correlation coefficient shows a good correlation of the predicted class memberships with the manually (‘truly’) observed ones. The 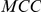 value of the second classification step (adheforent vs. non-adherent exterior conidia) is slightly lower with 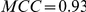. This is due to the difference in the number of adherent and non-adherent exterior conidia that gives rise to an imbalance of true positives and true negatives.

The statistical analysis of the phagocytosis ratio confirmed the results of previous experiments showing a significantly increased phagocytosis of the *pksP* mutant conidia in contrast to the wild type. This is due to an increased amount of ß1-3glucan displayed on the surface of resting *pksP* mutant conidia by loss of a functional melanin layer, thereby enhancing phagocytosis mediated by the C-type lectin-like surface receptor dectin-1 [32], [33]. Therefore, we demonstrate that our method yields correct image analysis compared with a manual procedure accompanied by a drastically reduced expenditure of time.

Furthermore, we prove that the *pksP* mutant conidia show beside an altered uptake ratio by macrophages increased conidial aggregation behaviour compared with the wild type. During germination, conidia undergo a drastic remodelling of the conidial cell wall. This includes loss of the outer rodlet/melanin cell wall layer, leading to the exposure of the inner cell wall layer. Fontaine *et al.* showed that during this process α1-3glucan becomes exposed on the conidial surface which is made responsible for the aggregation of swollen conidia [33]. Like ß1-3glucan, α1-3glucan is a major component of the inner cell wall and becomes accessible during germination [34]. Since resting *pksP* mutant conidia display an increased amount of ß1-3glucan exposed on the conidial surface, it is reasonable to assume that the accessibility of α1-3glucan on resting *pksP* mutant conidia is also higher than on wild-type conidia. In contrast to our results, Fontaine *et al.* did not observe an increased aggregation behaviour of the *pksP* mutant conidia. One possible explanation could be the different experimental setup. In our method, we investigated the phagocytosis of resting conidia, whereas Fontaine *et al.* determined the aggregation behaviour over a longer period of time. Therefore, the morphological differences in the conidial cell wall architecture between resting wild-type and *pksP* mutant conidia become less important due to the loss of the masking rodlet/melanin layer during germination. Furthermore, the method of aggregation measurement by Fontaine *et al.* was based on whole culture analysis, whereas our method detects individual cells and is more sensitive to morphological aspects.

In summary, the phagocytosis of *A. fumigatus* conidia by macrophages is a complex process that is affected by various parameters. Based on the full spatial information of pathogens and immune cells as well as all possible interrelations between them, the presented analysis reveals that *pksP* mutant conidia do not only have an increased phagocytosis ratio as compared to wild-type conidia but also show a significantly increased aggregation behaviour. Previous experimental setups did not allow for studying these morphological aspects in detail, since these can only be obtained as the result of analysing the behavior of individual cells in high-throughput microscopy measurements.

The developed ruleset for processing microscopic raw data is easy to implement and allows for the fully automated and context-based analysis of image data. The image analysis is suitable to obtain reproducible results from spatially resolved biological data and to replace tedious and error-prone manual work. High precision rates and classification scores show that this method has the potential to be used in high-throughput microscopy measurements and may be applied to other strains of *A. fumigatus* as well as to other fungal species. Since conidia or small round cells represent the infectious agents of many diseases, the understanding of the recognition and phagocytosis process can contribute to a better understanding of the virulence process of other relevant pathogens like *Candida* spp., *Cryptococcus neoformans* or *Histoplasma capsulatum*. Therefore, the developed ruleset will contribute to improve the quantity and quality of phagocytosis analyses in general. The fast analysis and statistical evaluation of features from large amounts of data are also required in the context of developing spatiotemporal *in silico* models of the host-pathogen interaction.

## Supporting Information

File S1 Documentation of the ruleset that was used in the automated image analysis by the software Definiens Developer XD.
(DOC)

File S2 Schematic representation of the analysis process.
(DOC)
