# Supplementary material for: Automated Image Analysis of the Host-Pathogen Interaction between Phagocytes and Aspergillus fumigatus
Source: PLoS One. 2011 May 5;6(5):e19591. doi: 10.1371/journal.pone.0019591 (PMC3088683; doi:10.1371/journal.pone.0019591)
Supplement: File S1 — Documentation of the ruleset that was used in the automated image analysis by the software Definiens Developer XD. (DOC) [file pone.0019591.s001.doc]

**Supporting Information S1.**

We provide the complete documentation of the ruleset that was used in the automated image analysis by the software Definiens Developer XD.

**Classes:**

_Temp1

_Temp2

_Temp3

adherentConidia

innerConidia

outlyingConidia

**Customized Features:**

Conidia_Sum: [No. of adherentConidia]+[No. of innerConidia]+[No. of outlyingConidia]

ration of temp3 to temp2 objects: [No. of _Temp3]/([No. of _Temp2]+[No. of _Temp3])

**Process: Main:**

Conidia Evaluation

- reset

delete layer: delete image layer 'filtered'

delete image object level: at New Level: delete

delete image object level: at Level 2: delete

delete layer: delete image layer 'distance map'

- Layer 2 - all Conidia
  - Layer Filtering and Segmentation

convolution filter: convolution filter (Gauss Blur, 3 x 3 x 1): 'Layer 2' => 'Gauss'

pixel min/max filter (prototype): pixel min\max filter (prototype) (5 x 1; Diff. center to darkest ): 'Gauss' =>
 'filtered'

automatic threshold: _threshold=auto threshold on filtered

multi-threshold segmentation: creating 'New Level': unclassified <= _threshold < _Temp1 on filtered

grow region: unclassified at New Level: <- _Temp1 Area <= 10

- - Simple Conidia - small, dark enclosed regions

pixel-based object resizing: _Temp1 at New Level: grow into unclassified with Area > 50

find enclosed by class: unclassified at New Level: enclosed by _Temp1: _Temp2 +

chessboard segmentation: _Temp1 at New Level: chess board: 1

pixel-based object resizing: loop: _Temp2 at New Level: grow into _Temp1, _Temp2 with Mean filtered
 ratio PPO(0) > 1 where rel. area of object pixels in (5 x 5) >=0.2

find enclosed by class: at New Level: enclosed by _Temp2: _Temp2 +

grow region: _Temp2 at New Level: <- _Temp2 Rel. border to _Temp2 = 1

pixel-based object resizing: 3x: _Temp2 at New Level: grow into all where rel. area of object pixels in
 (5 x 5)>=0.4

merge region: _Temp1 at New Level: merge region

_Temp2 with Area <= 10 at New Level

_Temp1 neighbour image object: do

update variable: temp = 0

_Temp2 neighbour image object: do

update variable: if with Area > 50 : temp = 1

assign class: if with temp = 0 : _Temp3

assign class: _Temp2 with Rel. border to _Temp3 > 0 at New Level: _Temp3

merge region: _Temp3 at New Level: merge region

pixel-based object resizing: 6x: _Temp3 at New Level: shrink using unclassified where rel. area of object
 pixels in (5 x 5) >=0.3

pixel-based object resizing: 2x: _Temp3 at New Level: grow into unclassified where rel. area of object
 pixels in (5x 5) >=0.3

assign class: _Temp2, _Temp3 with Area < 50 and Rel. border to _Temp2 = 0 at New Level: _Temp1

assign class: _Temp2, _Temp3 with Area < 100 and Rel. border to _Temp2 > 0 at New Level: _Temp1

- - false detected temp1 - small, completely bright regions

assign class: _Temp1 with Area >= 150 and Roundness <= 0.4 at New Level: _Temp2

- - false detected temp2

assign class: _Temp2, _Temp3 with Area > 380 at New Level: unclassified

- - Clean up

grow region: loop: unclassified at New Level: <- _Temp1

update variable: ration_temp3_temp2 = ration of temp3 to temp2 objects

assign class: _Temp2, _Temp3 at New Level: innerConidia

assign class: _Temp1 at New Level: unclassified

Quickmerge: Quickmerge(New Level,unclassified)

- Layer 1 - Outside Conidia
  - which Conidia have bright values on Layer 1

convolution filter: convolution filter (Gauss Blur, 3 x 3 x 1): 'Layer 1' => 'Gauss'

pixel min/max filter (prototype): pixel min\max filter (prototype) (5 x 1; Diff. center to darkest ): 'Gauss' =>
 'filtered_Layer1'

assign class: innerConidia with Mean Layer 1 > 30 at New Level: outlyingConidia

- Layer 3 - Macrophages and Adherent Conidia
  - reset

delete layer: delete image layer 'filtered'

convolution filter: convolution filter (Gauss Blur, 3 x 3 x 1): 'Layer 3' => 'Gauss'

pixel min/max filter (prototype): pixel min\max filter (prototype) (5 x 1; Diff. center to darkest ): 'Gauss' => 'filtered_Layer3'

automatic threshold: _threshold=auto threshold on filtered

multi-threshold segmentation: unclassified at New Level: unclassified <= _threshold < _Temp1 on filtered

pixel-based object resizing: _Temp1 at New Level: grow into unclassified

merge region: _Temp1 at New Level: merge region

grow region: unclassified at New Level: <- _Temp1 Area < 40

- - Adherent Conidia
    - outlyingConidia at New Level

assign class: _Temp1 with Area > 250 neighbour image object: _Temp3

assign class: _Temp1 with Distance to scene border = 0 Pxl and Area <= 250 neighbour image
 object: _Temp3

assign class: if with Rel. border to _Temp3 > 0 : adherentConidia

- - Clean up

assign class: _Temp3 at New Level: _Temp1

- wrongly classified conidia

assign class: _Temp3 at New Level: unclassified

remove objects: _Temp1 with Area <= 5 at New Level: remove objects into _Temp1, adherentConidia,
 innerConidia, outlyingConidia, unclassified

find enclosed by class: unclassified with Area > 450 at New Level: enclosed by _Temp1, adherentConidia,
 innerConidia, outlyingConidia: _Temp3 +

assign class: outlyingConidia with Rel. border to _Temp3 > 0 at New Level: innerConidia

assign class: outlyingConidia with Rel. border to _Temp1 > 0 at New Level: adherentConidia

- export
  - statistics - area, enclosed area ... of conidia and macrophages, ratios ...

export object statistics: adherentConidia, innerConidia, outlyingConidia at New Level: export object
 statistics

export project statistics: export project statistics

- - clumped objects - assign clump IDs

pixel-based object resizing: adherentConidia, innerConidia, outlyingConidia at New Level: grow into all

update variable: counter = 0

update variable: adherentConidia, innerConidia, outlyingConidia at New Level: clumpID = counter

adherentConidia, innerConidia, outlyingConidia at New Level

update variable: clumpID = counter

adherentConidia, innerConidia, outlyingConidia neighbour image object: do

update variable: clumpID = counter

adherentConidia, innerConidia, outlyingConidia neighbour image object: do

update variable: clumpID = counter

adherentConidia, innerConidia, outlyingConidia neighbour image object: do

update variable: clumpID = counter

adherentConidia, innerConidia, outlyingConidia neighbour image object: do

update variable: clumpID = counter

adherentConidia, innerConidia, outlyingConidia neighbour image object: do

update variable: clumpID = counter

adherentConidia, innerConidia, outlyingConidia neighbour image object: do

update variable: clumpID = counter

adherentConidia, innerConidia, outlyingConidia neighbour image object: do

update variable: clumpID = counter

update variable: counter += 1

- - export object statistics: adherentConidia, innerConidia, outlyingConidia at New Level: export object statistics
