# Supplementary material for: Automated Image Analysis of the Host-Pathogen Interaction between Phagocytes and Aspergillus fumigatus
Source: PLoS One. 2011 May 5;6(5):e19591. doi: 10.1371/journal.pone.0019591 (PMC3088683; doi:10.1371/journal.pone.0019591)
Supplement: File S2 — Schematic representation of the analysis process. (DOC) [file pone.0019591.s002.doc]

**Supporting Information S2.**

Input:

Images

Filtering:

Gauss Filter

edge detection

automatic threshold

Segmentation:

multiresolution segmentation

find bright areas or dark areas enclosed by bright objects using cell area threshold A_M_ and A_C_ and roundness parameter ρ

segmented conidia objects

Classification:

based on intensity threshold I_C_

classified conidia objects:

phagocytosed

adherent

exterior

Export:

for each conidia object:

class membership

area

number of neighbours

for each image:

number of objects per class

Statistical Analysis:

for each image:

calculation of phagocytosis ratio

calculation of adhesion ratio

calculation of number of clusters and size of each cluster

manual determination of TP, FP, FN, TN

for each replicate:

calculation of mean and standard deviation of phagocytosis ratio, adhesion ratio and number of clusters

for each strain:

calculation of mean and standard deviation of phagocytosis ratio, adhesion ratio and number of clusters

graphical plot for phagocytosis ratio, adhesion ratio and number of clusters

for all images:

calculation of precision P, sensitivity S, F_1_-measure and MCC-score
